# Low HLA binding of diabetes-associated CD8+ T-cell epitopes is increased by post translational modifications

**DOI:** 10.1186/s12865-018-0250-3

**Published:** 2018-03-21

**Authors:** John Sidney, Jose Luis Vela, Dave Friedrich, Ravi Kolla, Matthias von Herrath, Johnna D. Wesley, Alessandro Sette

**Affiliations:** 1 La Jolla Institute for Allergy and Immunology, La Jolla, CA 92130 USA; 2 Novo Nordisk Research Center Seattle, Inc., 530 Fairview Ave N, Seattle, WA 98109 USA

**Keywords:** Diabetes, Insulin, T cell epitopes, Class I MHC, HLA-peptide binding affinity

## Abstract

**Background:**

Type 1 diabetes (T1D) is thought to be an autoimmune disease driven by anti-islet antigen responses and mediated by T-cells. Recent published data suggests that T-cell reactivity to modified peptides, effectively neoantigens, may promote T1D. These findings have given more credence to the concept that T1D may not be solely an error of immune recognition but may be propagated by errors in protein processing or in modifications to endogenous peptides occurring as result of hyperglycemia, endoplasmic reticulum (ER) stress, or general beta cell dysfunction. In the current study, we hypothesized that diabetes-associated epitopes bound human leukocyte antigen (HLA) class I poorly and that post-translational modifications (PTM) to key sequences within the insulin-B chain enhanced peptide binding to HLA class I, conferring the CD8+ T-cell reactivity associated with T1D.

**Results:**

We first identified, through the Immune Epitope Database (IEDB; www.iedb.org), 138 published HLA class I-restricted diabetes-associated epitopes reported to elicit positive T-cell responses in humans. The peptide binding affinity for their respective restricting allele(s) was evaluated in vitro. Overall, 75% of the epitopes bound with a half maximal inhibitory concentration (IC50) of 8250 nM or better, establishing a reference affinity threshold for HLA class I-restricted diabetes epitopes. These studies demonstrated that epitopes from diabetes-associated antigens bound HLA with a lower affinity than those of microbial origin (binding threshold of 500 nM for 85% of the epitopes). Further predictions suggested that diabetes epitopes also bind HLA class I with lower affinity than epitopes associated with other autoimmune diseases. Therefore, we measured the effect of common PTM (citrullination, chlorination, deamidation, and oxidation) on HLA-A*02:01 binding of insulin-B-derived peptides, compared to native peptides. We found that these modifications increased binding for 44% of the insulin-B epitopes, but only 15% of the control peptides.

**Conclusions:**

These results demonstrate that insulin-derived epitopes, commonly associated with T1D, generally bind HLA class I poorly, but can be subject to PTM that improve their binding capacity and may, in part, be responsible for T-cell activation in T1D and subsequent beta cell death.

**Electronic supplementary material:**

The online version of this article (10.1186/s12865-018-0250-3) contains supplementary material, which is available to authorized users.

## Background

Type 1 diabetes (T1D) is characterized by the presence of autoantibodies and islet beta cell loss leading to metabolic dysfunction and hyperglycemia. Beta cell loss is, in part, mediated by T-cells that are reactive to insulin-derived epitopes [1]. Recent studies have indicated that processing defects in proinsulin may lead to antigen processing errors or altered expression of proinsulin-derived epitopes. Additionally, the distinctive inflammation in the pancreas may trigger increased expression of enzymes, such as tissue transglutimase (tTG), or other mechanisms leading to post-translational modifications (PTMs) of native epitopes, generating peptides with greater binding affinity to HLA, and enhancing T-cell recognition and activation and increased beta cell death [2]. Responding T-cells would have appropriately passed through negative selection in the thymus by recognizing weakly immunogenic native epitopes and migrated into the periphery. Then, in the periphery, in T1D-susceptible individuals, PTM of epitopes during antigen processing leads to a higher affinity to HLA, allowing T-cells to recognize the peptide-HLA (pHLA) complex and undergo activation and expansion, rather than anergy. This then leads to pancreatic inflammation and beta cell death.

The polymorphisms of HLA class II genes are major risk factors for T1D and CD4 T-cells are widely studied and continue to be of high interest. Human CD4 T-cells recognizing a modified epitope from the insulin-A chain were first described in the context of HLA DR4 [3]. Another study demonstrated activation of CD4 T-cells from a recent-onset T1D patient in response to a modified preproinsulin-derived epitope [4]. Additionally, in a recent study from DeLong et al. [5], CD4 T-cells reactive to epitopes from fused peptides were found in insulitic lesions in T1D. Though cytolytic CD8 T-cells (CTLs) have been seen in the pancreatic infiltrate and diabetes antigen-specific cells can be detected in the periphery, HLA class I and CD8 T-cell-pHLA interactions have not been as broadly investigated in T1D [6].

We hypothesized that CTLs recognizing diabetes epitopes could escape negative selection in the thymus by having only weak HLA class I binding capacity. The present study was designed to systematically address the issue of HLA binding affinity of human class I-restricted epitopes derived from diabetes-associated antigens. Accordingly, we experimentally determined the HLA class I binding capacity of epitopes from diabetes-associated proteins that were reported to illicit positive CTL responses in humans with T1D. Further, utilizing bioinformatic predictions, we compared the class I binding patterns of diabetes epitopes with those of non-diabetes autoimmune disease and viral epitopes. Finally, we experimentally tested whether PTM modification by citrullination, chlorination, deamidation, and oxidation of insulin epitopes could increase peptide binding to HLA-A*02:01, the HLA class I allele most commonly studied in the context of T1D is HLA-A*02:01, and thereby potentially leading to a stronger in vivo T-cell response.

## Results

### Diabetes-associated CTL epitopes in the published literature

To identify diabetes-associated CTL epitopes from the published literature, we employed a bioinformatics tool recently developed in the Sette laboratory [7]. This tool automatically extracts relevant data from the Immune Epitope Database (IEDB; www.iedb.org) and generates reference sets of validated epitopes from various disease indications. Here, the tool was applied to epitopes in the IEDB that are derived from a set of human proteins associated with diabetes [8], further modified to take advantage of the IEDB’s search interface that allows for identification of epitope records directly associated with studies related to a specific disease and/or autoimmune context.

Accordingly, using the IEDB disease finder, the query was configured to include epitopes derived from antigens associated with T1D, pre-diabetes, and diabetes mellitus studies (Additional file 1). The query was also structured for epitopes reported to elicit positive T-cell responses in human hosts as determined using multimer/tetramer staining assays or readouts based on intracellular cytokine staining (ICS), enzyme-linked immunospot (ELISPOT), or ^51^Cr-release assays. Responses induced following either in vitro or ex vivo stimulation were allowed. Finally, only epitopes between 8 and 11 residues in length were considered, agreeing with the most canonical peptide sizes bound by class I molecules. This generated a set of 138 epitopes (Additional file 2). Notably, 114 of the 138 epitopes (83%) were restricted by HLA-A*02:01 or A2 serological specificities. As A*02 alleles are the most common in almost all major ethnic/geographic populations worldwide [9, 10] and, as a result, have been the most extensively studied, it is unlikely that this bias is related to diabetes incidence.

### HLA class I binding capacity of T1D-associated CTL epitopes

The 138 peptides were tested for their capacity to bind their respective HLA class I restricting allele(s) in classical competition assays based on the inhibition of the binding of high affinity radiolabeled ligands to purified major histocompatibility (MHC) molecules, as described in the Methods. In instances where the precise restriction was not available, binding to the most common representative subtype from the same allele family was assayed (e.g., A*02:01 for A02; B*07:02 for B7; A*03:01 for A03, etc.). The affinity of each epitope to its reported HLA restricting allele(s) is shown in Table 1. For epitopes reported to be restricted by multiple alleles, each restriction is shown separately. The different alleles assayed, a tally of the number of corresponding peptides tested, and the number that were considered high or intermediate binders, are provided in Table 2.

**Table 1:** HLA class I binding of CTL epitopes associated with previously defined diabetes-associated proteins

Source protein	Epitope sequence	Target assay	IC50 nM
AN1-type zinc finger protein 5	SASVQRADTSL	B*07:02	11
Bruton agammaglobulinemia tyrosine kinase	CLCLLNPQGT	A*02:01	–
	HLASEKVYAI	A*02:01	4064
	KLANIQCLCL	A*02:01	97
	KLANIQCPCL	A*02:01	121
	LASEKVYAI	A*02:01	6926
	SLTAISTTL	A*02:01	242
	SLTTISTTL	A*02:01	237
	YIPSCTVVGM	A*02:01	9506
Fms-related tyrosine kinase 3	KVLHELFGMDI	A*02:01	11
	VLHELFGMDI	A*02:01	287
Fms-related tyrosine kinase 3 ligand	ALARGAGTVPL	A*02:01	29
	SMPQGTFPV	A*02:01	631
Glial fibrillary acidic protein isoform 2	NLAQDLATV	A*02:01	67
	QLARQQVHV	A*02:01	–
	SLEEEIRFL	A*02:01	472
Glutamate decarboxylase 2	RFKMFPEVK	A*11:01	18,296
	MFPEVKEKG	A*11:01	–
	SPGSGFWSF	B*07:02	1173
	TSEHSHFSL	B*35:01	–
	ELAEYLYNI	A*02:01	637
	FLQDVMNIL	A*02:01	70
	ILMHCQTTL	A*02:01	478
	LLQEYNWEL	A*02:01	35
	RMMEYGTTMV	A*02:01	709
	VMNILLQYV	A*02:01	2024
	VMNILLQYVV	A*02:01	2308
	ACDGERPTL	B*07:02	28,719
	AHVDKCLEL	B*07:02	–
	APVIKARMM	B*07:02	18,197
	HPRYFNQLST	B*07:02	1252
	IPSDLERRIL	B*07:02	3858
Heat shock 70 kDa protein 1	LLDVAPLSL	A*02:01	51
	LLLLDVAPL	A*02:01	10
	LMGDKSENV	A*02:01	7374
Heat shock 70 kDa protein 6	FIQVYEVERA	A*02:01	7024
	FMTSSWWGA	A*02:01	378
	FMTSSWWRA	A*02:01	101
	FMTSSWWRAPL	A*02:01	84
	GIPPAPHGV	A*02:01	219
	GLLQVHHSCPL	A*02:01	5.8
	GVFIQVYEV	A*02:01	460
	KCQEVLAWL	A*02:01	–
	LLGRFELIGI	A*02:01	233
	LLHVHHSCPL	A*02:01	7839
	LLQVHHSCPL	A*02:01	3087
	NLLGRFELI	A*02:01	764
	NLLGRFELIGI	A*02:01	1.8
	SLASLLPHV	A*02:01	9.1
	SMCRFSPLTL	A*02:01	1149
	SVASLLPHV	A*02:01	1758
	VLNSLASLL	A*02:01	4968
	VLNSVASLL	A*02:01	1143
	VLVEGSTRI	A*02:01	17,161
Heat shock 70 kDa protein 6 variant	SLFEGVDFYT	A*02:01	51
Heat shock 70 kDa protein 1A variant	GIPPAPRGV	A*02:01	1226
	LIFDLGGGT	A*02:01	5416
Heat shock protein HSP 90-beta	ILDKKVEKV	A*02:01	11,237
Insulin	WGPDPAAA	A*02:01	–
	GIVEQCCTSI	A*02:01	7446
	LCGSHLVEAL	A*02:01	–
	SHLVEALYLV	A*02:01	2153
	ALWGPDPAAA	A*02:01	1008
	HLVEALYLV	A*02:01	134
	SLYQLENYC	A*02:01	16,923
	RLLPLLALL	A*02:01	145
		A*24:02	10,212
	VCGERGFFYT	A*01:01	–
		A*02:01	–
		B*08:01	–
		B*18:01	626
	ALWMRLLPLL	A*02:01	190
		A*24:02	6181
		B*08:01	33
	ALWMRLLPL	A*02:01	218
		B*08:01	107
	GSHLVEALY	A*01:01	30,269
	LVCGERGFFY	A*01:01	–
		A*03:01	27,363
		A*11:01	27,207
	GERGFFYT	A*01:01	–
		B*08:01	1377
	LALWGPDPAA	A*02:01	30,300
	RLLPLLALLAL	A*02:01	109
	HLCGSHLVEA	A*02:01	7536
	SLQKRGIVEQ	A*02:01	–
	LYLVCGERGF	A*24:02	5255
	LWMRLLPLL	A*24:02	545
	ALWGPDPAAAF	A*01:01	–
		A*24:02	15,101
	ERGFFYTPK	A*03:01	–
	PLALEGSLQK	A*03:01	–
	PLLALLALWG	A*03:01	–
	ALYLVCGER	A*03:01	2793
		A*11:01	28,341
	SLQPLALEG	A*02:01	–
		A*03:01	–
	LPLLALLAL	B*07:02	610
		B*35:01	12,300
		B*51:01	1164
	WMRLLPLLAL	B*07:02	2359
	FYTPKTRRE	B*08:01	13,033
Islet amyloid polypeptide precursor	FLIVLSVAL	A*02:01	26
	KLQVFLIVL	A*02:01	1372
Islet-specific glucose-6-phosphatase	FLWSVFMLI	A*02:01	26
Islet-specific glucose-6-phosphatase isoform 1	FLWSVFWLI	A*02:01	55
	RLLCALTSL	A*02:01	91
	LNIDLLWSV	A*02:01	684
	VLFGLGFAI	A*02:01	149
	NLFLFLFAV	A*02:01	1228
	YLLLRVLNI	A*02:01	57
	FLFAVGFYL	A*02:01	1.1
Protein tyrosine phosphatase	LLPPLLEHL	A*02:01	444
	SLAAGVKLL	A*02:01	3686
	SLSPLQAEL	A*02:01	101
	ALTAVAEEV	A*02:01	1306
	SLYHVYEVNL	A*02:01	209
	TIADFWQMV	A*02:01	4693
	VIVMLTPLV	A*02:01	2117
	MVWESGCTV	A*02:01	126
Tyrosine-protein kinase BTK	LASEKVYTI	A*02:01	1254
Tyrosine-protein kinase Lyn isoform B	LMFWSPSHSCA	A*02:01	143
	RLQREWHTL	A*02:01	1230
Tyrosine-protein phosphatase non-receptor type 11	RLGPVARTRV	A*02:01	581
	STVASRLGPV	A*02:01	25,069
	STVASWLGPV	A*02:01	9227
	TLSSRVCCRT	A*02:01	2784
	TVASRLGPV	A*02:01	7695
Zinc finger protein 36, C3H1 type-like 2	GLPAGAAAQA	A*02:01	530
	HLSYHRLLPL	A*02:01	4259
	HLSYHWLLPL	A*02:01	3817
	RLLPLWAAL	A*02:01	52
	RLLPLWAALPL	A*02:01	5.4
	RLRPLCCTA	A*02:01	8234
	WLLPLWAAL	A*02:01	4.2
	WLLPLWAALPL	A*02:01	3.2
Zinc transporter 8 isoform a	ALGDLFQSI	A*02:01	76
	AVAANIVLTV	A*02:01	575
	FLLSLFSLWL	A*02:01	16
	HIAGSLAVV	A*02:01	216
	ILAVDGVLSV	A*02:01	2.2
	ILKDFSILL	A*02:01	39
	ILVLASTITI	A*02:01	675
	IQATVMIIV	A*02:01	9043
	KMYAFTLES	A*02:01	880
	RLLYPDYQI	A*02:01	124
	SISVLISAL	A*02:01	1555
	TMHSLTIQM	A*02:01	104
	VAANIVLTV	A*02:01	320
	VVTGVLVYL	A*02:01	9.0
	LLIDLTSFL	A*02:01	8.1
	LLSILCIWV	A*02:01	734
	LLSLFSLWL	A*02:01	152

A dash indicates IC50 >40,000 nM

**Table 2:** HLA class I tested, and epitope binding rates

Target assay	n	High affinity	% high	Int. affinity	% int.	Total binders	% binders
A*01:01	5	0	0.0	0	0.0	0	0.0
A*02:01	115	56	48.7	34	29.6	90	78.3
A*03:01	6	0	0.0	1	16.7	1	16.7
A*11:01	4	0	0.0	0	0.0	0	0.0
A*24:02	5	0	0.0	1	20.0	1	20.0
B*07:02	9	1	11.1	5	55.6	6	66.7
B*08:01	5	2	40.0	1	20.0	3	60.0
B*18:01	1	0	0.0	1	100.0	1	100.0
B*35:01	2	0	0.0	0	0.0	0	0.0
B*51:01	1	0	0.0	1	100.0	1	100.0
Total	153	59	38.6	44	28.8	103	67.3

In total, 56 (48.7%) of the 115 A*02:01-restricted epitopes bound with high affinity (IC50 < 500 nM); another 34 (29.6%) bound with intermediate affinity (IC50 in the 500–5000 nM range). Of the 38 non-A*02 restrictions, only 3 (7.9%) were associated with an affinity of 500 nM or better and another 10 (26.3%) bound with only intermediate affinity. Thus, overall, 103 of 153 (67.3%) of the HLA/epitope combinations were associated with binding at the 5000 nM, or better, level; that is, 59 (38.6%) were associated with high affinity, and another 44 (28.8%) with intermediate affinity.

**Fig. 1:**
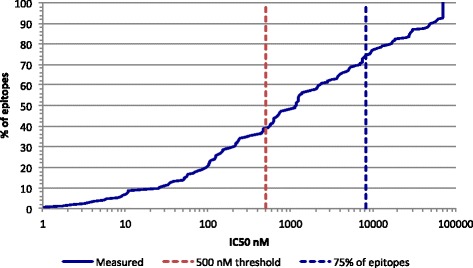
HLA class I binding capacity of diabetes-associated CTL epitopes: A cumulative percentage of diabetes associated epitopes is plotted as a function of the binding capacity of the epitopes assayed for their corresponding HLA class I restricting allele. As shown, about 40% of the epitopes bound with an affinity of 500 nM (red dashed line), or better, and 75% bound at the 8250 nM level (blue dashed line)

In terms of thresholds, 75% of the epitopes bound with an IC50 of 8250 nM or better, and 90% of the epitopes bound with an IC50 of 48,000 nM (Fig. 1). These results established a reference threshold for binding affinities of HLA class I-restricted diabetes epitopes, and confirmed that the majority of diabetes epitopes bound with detectable affinity to HLA, consistent with HLA binding being a necessary (albeit not sufficient) requisite for HLA class I-specific immunogenicity.

### Comparative HLA class I binding capacity of diabetes-associated CTL epitopes

**Fig. 2:**
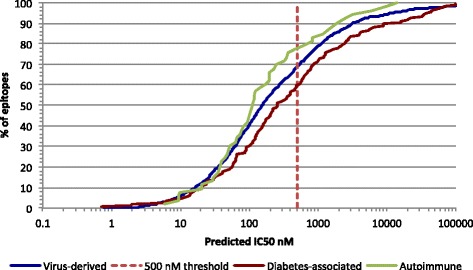
Binding capacity of various types of class I epitopes: The predicted binding affinity of diabetes-associated epitopes (red line) is compared with the predicted binding capacity of virus derived epitopes (blue line), and epitopes associated with other autoimmune diseases (green line). The 500 nM threshold (dashed red line) identifies 60%, 70%, and 78% of the respective epitopes

The binding threshold identified above is notable in that it is somewhat higher (i.e., associated with lower affinity) than the binding threshold (500 nM) previously found to be associated with the vast majority (> 80%) of HLA class I-restricted T-cell epitopes derived from various pathogens [11–13]. To more directly compare diabetes-associated epitopes to those of pathogenic origin, we next utilized a bioinformatic approach used previously [13] and generated predicted binding affinities for each diabetes-associated epitope to its restricting allele(s) utilizing the SMM algorithm hosted by the IEDB analysis resource. Predictions were also generated for a large panel of over 2200 virus-derived epitopes identified in the IEDB. The cumulative predicted affinity of the two epitope sets were then compared (Fig. 2). As shown, the virus-derived epitopes were predicted to bind at a higher rate than the diabetes-associated epitopes, where a predicted binding threshold of 500 nM captured about 70% of the virus epitopes, but only 60% of the diabetes epitopes. Similarly, 75% of the virus epitopes were expected to bind at 750 nM or better, in comparison to 1260 nM for the diabetes epitopes. The results for the viral epitopes were consistent with previous reports where a binding threshold of 500 nM was associated with approximately 85% of epitopes [11, 12]. These findings indicate that the overall binding affinity is lower for diabetes-associated T-cell epitopes when compared to virus-derived peptides.

These observations were not entirely surprising, as it has been hypothesized that self-epitopes might bind with reduced affinity, and that this is likely characteristic of autoimmunity in general (see, e.g., [2, 14]). However, when we evaluated the predicted binding affinity of 53 non-diabetes autoimmune epitopes retrieved from the IEDB using identical methodology, we found that these epitopes were not only predicted to bind better than diabetes-associated peptides, but even better than the viral epitopes. In fact, a 500 nM threshold identifies 80% of the selected non-diabetes autoimmune-associated epitopes, with 75% of them predicted to bind at the 355 nM level or better (Fig. 2).

### Selection of post-translationally modified peptides

The measured and predicted lower affinity of diabetes epitopes could be explained if the epitopes recognized by diabetes-associated T-cells are often PTM products, and the modification is associated with increased HLA binding. To test this hypothesis, which was also previously suggested by McGinty and James [15], we analyzed the binding affinity of diabetes-associated epitopes, specifically from the insulin-B 30-mer, and control non-epitopes, modified by common PTMs, including citrullination, oxidation, deamidation, and chlorination. In each case, as described following, sets of all possible overlapping 9- or 10-mer sequences incorporating the various modifications were synthesized.

In terms of oxidation, Strollo et al. [16] using sodium dodecyl sulfate polyacrylamide gel electrophoresis (SDS-PAGE), three-dimensional fluorescence, and mass spectrometry (MS) demonstrated that histidine (His5), cysteine (Cys7), and L-phenylalanine (Phe24) residues could be oxidized in insulin-B (corresponding to positions 29, 31, and 49, respectively, in the full unspliced insulin sequence). To address the impact of oxidation of these residues on pHLA binding, 66 peptides were synthesized (see Additional file 3), comprising 14 peptides with oxidized Cys7; 14 with oxidized Phe24; and 38 with all residues oxidized (i.e., pan oxidation; see Methods).

Strollo et al. also reported that the tyrosines (Tyr) in positions 16 and 26 in the insulin B-chain (positions 40 and 50 in the unspliced sequence) were chlorinated. Therefore, another 29 peptides were synthesized to evaluate chlorination of both tyrosine residues.

Further, deamination of asparagine (Asn) and glutamine (Gln) residues have been described [4]. Deamination of Asn or Gln leads to and aspartic acid (Asp) or glutamic acid (Glu), respectively, or isoaspartic and isoglutamic acid, respectively. To evaluate the impact of Asn or Gln deamination of insulin B-chain eptiopes, we synthesized another 16 peptides.

In addition to the various published PTMs of the B-chain, we also investigated the effect of citrullination throughout the entire unspliced insulin sequence, which required the synthesis of 70 peptides. Collectively, a total of 276 peptides were synthesized, comprising 181 modified peptides and the corresponding 95 unmodified 9- and 10-mers. All peptides were evaluated for binding to HLA-A*02:01, as described in the Methods. The HLA-A*02:01 binding of each modified peptide is listed, along with its cognate unmodified (i.e., wild type, WT) peptide, in Additional file 3. The location of the various residues subjected to modification in the full-length insulin sequence is shown in Additional file 4.

### HLA-A*02:01 binding of PTM insulin-derived peptides

We next evaluated the impact of these PTMs on the HLA-A*02:01 binding capacity of insulin-derived peptides, including nine that were previously reported as A*02:01 restricted T cell epitopes (see Additional file 3). Of the nine previously reported epitopes, four (44%) were associated with a PTM-dependent improvement of affinity of at least two-fold (average = 3.76 +/− 2.73-fold) compared to the WT peptide (Table 3, top 4 epitopes). These improved peptides include the leader sequence 2–10 9-mer and 2–11 10-mer epitopes; here, modification of the arginine (Arg) in position 6 by citrullination improved binding of the 9-mer and 10-mer 7.83- and 2.76-fold, respectively. Two peptides (one 9- and one 10-mer) from the 33–42 region of the beta chain with chlorination of Tyr40 bound 2- to 2.5-fold better than the corresponding WT sequences. Overall, because in several cases multiple modifications of each epitope were tested, 26.7% (4/15) of the total modifications to known insulin-derived A*02:01-restricted T-cell epitopes resulted in a peptide with increased binding.

**Table 3:** Insulin-derived epitope-HLA-A*02 binding was improved by PTMs

				Sequence			A*02:01 binding		Fold increase
Set	Start	Segment	Len	WT	Modified	Modification	WT	Modified	
Improved A*02 epitopes	2	Leader	9	ALWMRLLPL	ALWMULLPL	Citrullination	218	28	7.83
			10	ALWMRLLPLL	ALWMULLPLL	Citrullination	190	69	2.76
	33	B chain	10	SHLVEALYLV	SHLVEALJLV	Chlorination	2153	1066	2.02
	34	B chain	9	HLVEALYLV	HLVEALJLV	Chlorination	134	55	2.43
Other improved peptides	1	Leader	9	MALWMRLLP	MALWMULLP	Citrullination	4539	2117	2.14
	1	Leader	10	MALWMRLLPL	MALWMULLPL	Citrullination	1255	262	4.79
	3	Leader	10	LWMRLLPLLA	LWMULLPLLA	Citrullination	406	61	6.62
	4	Leader	10	WMRLLPLLAL	WMULLPLLAL	Citrullination	654	310	2.11
	28	B chain	9	QHLCGSHLV	EHLCGSHLV	Deamidation	–	7237	> 5
	31	B chain	10	CGSHLVEALY	BGSHLVEALY	Oxidation of C	33,691	8043	4.19
	37	B chain	10	EALYLVCGER	EALYLVCGEU	Citrullination	10,069	3100	3.25
	38	B chain	10	ALYLVCGERG	ALJLVCGERG	Chlorination	8784	2734	3.21
	39	B chain	9	LYLVCGERG	LYLVCGEUG	Citrullination	28,006	12,493	2.24
	40	B chain	9	YLVCGERGF	JLVCGERGF	Chlorination	6742	3163	2.13
					YLVBGERGY	Pan oxidation	6742	3244	2.08
	45	B chain	10	ERGFFYTPKT	ERGYYYTPKT	Pan oxidation	–	5730	> 7
	46	B chain	9	RGFFYTPKT	RGYYYTPKT	Pan oxidation	–	8805	> 4
	80	C peptide	10	LALEGSLQKR	LALEGSLQKU	Citrullination	–	16,354	> 2

A dash indicates IC50 > 40,000 nM

In addition to these previously published PTM epitopes, another 13 peptides (Table 3, bottom 13, below line) were found to be associated with improved binding as a result of PTM, compared to the unmodified counterpart. (For the 9-mer B-chain sequence, YLVCGERGF, 2 different modifications led to increased binding). However, only one of the 14 modified peptides — a citrullinated leader sequence peptide —bound with an affinity < 100 nM. By comparison, three of the four modified known HLA-A*02:01 restricted epitopes bound with affinities < 100 nM. The difference in the rate of improvement seen between these two peptide sets is significant (*p* = 0.0147).

Additional analyses evaluated the effect of 166 modifications of 86 control non-epitopes (or epitopes restricted by non-A*02 alleles). For these control peptides, improved binding (i.e., > 2-fold increase in affinity) was found for only 14 of the 166 modifications (8.4%); this corresponded to only 13/86 (15%) unique control peptides acquiring higher affinity following modification. The difference in rate of improvement, compared to the A*02-restricted insulin-B-derived epitope set, was significant (*p* = 0.05).

The effects of specific modifications on the binding of A*02 epitopes, non-epitopes, and in total, are summarized in Table 4. With respect to the epitopes, 2/3 (67%) sequences subjected to chlorination, and 2/5 (40%) modified by citrullination, resulted in improved binding. Oxidation did not result in improved binding in any of the seven cases examined. Taken together, however, it should be noted that the number of events probed here are not sufficient to make statements regarding statistical significance of differences between the different modifications. Further, because Asn or Gln residues were not present in the respective epitopes, none of the corresponding peptide sequences were subject to deamidation.

**Table 4:** Summary of effects of specific PTMs on HLA A*02:01 binding capacity

	Epitopes		Non-epitopes		Total	
Modification	n	% improved	n	% improved	n	% improved
Chlorination	3	66.7	26	7.7	29	13.8
Citrullination	5	40.0	65	10.8	70	12.9
Deamidation	0	–	16	6.3	16	6.3
Oxidation	7	0.0	59	6.8	66	6.1
Total	15	26.7	166	8.4	181	9.9

## Discussion

The role of T-cells in T1D has been demonstrated in mouse models and supported by the presence of CD4+ and CD8+ T-cells in human pancreata. Further, with the use of multimer technology and functional assays, inflammatory T-cells responsive to pancreas-derived antigens can be detected in peripheral blood samples from T1D subjects. Notably, these autoreactive T-cells have also been found in healthy, non-diabetic individuals, suggesting that such T-cells commonly make it through thymic selection and enter the peripheral tissue with little consequence to the individual. Peripheral modifications (PTM, mutation, processing defects) of native epitopes that are weakly to non-immunogenic in the thymus could induce unanticipated T-cell activation in the periphery. This would imply that T1D is less an error of the immune system and more the result of peripheral tissue dysfunction. Recent publications demonstrating the presence of hybrid and modified peptides, as well as reactive T-cells, in T1D+ subjects provides additional evidence to support this idea [17].

The present analyses assessed the capacity of 138 diabetes-associated CTL epitopes identified in the published literature to bind their reported HLA class I restricting allele(s). The data generated established a binding affinity reference threshold of 8250 nM that captures 75% of HLA class I-restricted diabetes epitopes, representing an affinity threshold somewhat lower than observed for pathogen-derived epitopes and predicted for epitopes from non-T1D autoimmune diseases. Notably, only 38.6% (59/138) of the diabetes epitopes studied bound at least one of its reported restriction elements with high affinity (IC50 < 500 nM) and another 28.8% (44/138) bound at least one allele with intermediate affinity (IC50 in the 500–5000 nM range). A generally lower rate of binding was associated with non-A2 alleles, possibly related to the fact these alleles have been less studied, and therefore the dataset might be inherently less representative and associated with reduced accuracy in terms of HLA restriction determination. It is also possible, as reported in a previous study [13], that different HLA alleles might be associated with epitope repertoires of differing breadth. Abreu and colleagues have similarly found that preproinsulin epitopes tend to be associated with low HLA class I (A02) binding affinity [2].

Intriguingly, from our data it appears that the lower overall affinity of diabetes-associated epitopes might be unique to this indication, as lower binding affinity thresholds do not extend to other autoimmune epitopes. The reasons for these apparent differences are not clear, but might be related to inherent differences in the immune response associated with T1D progression compared to multiple sclerosis and rheumatoid arthritis, the two indications from which the other autoimmune epitopes evaluated in the current study were derived.

To evaluate the potential impact of common PTMs on the HLA class I binding capacity, we synthesized a large set of insulin-derived A*02:01-restricted T-cell epitopes, and also control peptides derived from human insulin but not associated with T1D, incorporating various modifications, including citrullination, chlorination, deamidation, and oxidation. We found that these modifications increased binding for 44% of the known T-cell epitopes, but only 15% of the control peptides. This data supports the hypothesis that the epitopes recognized by diabetes-associated T-cells are often PTM products, and these modified epitopes are associated with increased HLA binding in peripheral tissue. Further study is required to understand the exact contribution of PTMs in T1D disease onset and to ascertain if they initiate disease, are a by-product of disease, or play a role in progression, and also to evaluate if they can be developed diagnostically to identify pathogenic T cells or clinically as a therapeutic.

It will be important for future studies to establish the generality of these findings by testing epitopes restricted by other HLA molecules. Query of the IEDB revealed that at least a dozen insulin-derived epitopes are restricted by other, non-A*02, alleles. The analyses could also be expanded to include HLA class II molecules, since posttranslational modifications (and citrullination in particular) have been discussed as a potential factor in modulating autoreactivity in rheumatoid arthritis and other autoimmune pathologies. Further, the present study provides a model to extend a similar analysis towards other antigens associated with T1D, such as GAD, HSP-70, IA-2, and IGRP.

It will also be crucial to test the modified epitopes associated with increased binding for recognition using PBMCs from at-risk, diabetic, and non-diabetic individuals. We have herein identified modified peptides that are high affinity A*02:01 binders, and could represent novel epitopes. It is of obvious interest to include these potential new epitopes in experiments using HLA-A*02:01^+^ PBMC from healthy, at-risk, and T1D^+^ donors for functional assessments, including T-cell proliferation and cytokine production. Parallel experiments could address similar analyses of insulin peptides, both native and post-translationally modified, that bind the K^d^ and D^b^ class I expressed by NOD mice to allow for a detailed in vivo evaluation of disease relevance. Further experiments could also start to address whether similar findings can be extended to insulin peptides that bind HLA class II, and in particular utilizing the diabetes associated DQ8 molecule.

Clearly, our understanding of the functional role of T-cells and HLA in T1D development and progression is continually evolving. Overall, these findings provide further evidence that human T1D is not solely the result of autoimmunity and may be driven by immune responses to neoantigens, generated from protein processing defects and metabolic dysregulation leading to cell stress.

## Conclusions

The present study experimentally and bioinformatically assessed the MHC binding capacity of HLA class I restricted T cell epitopes to demonstrate that T1D-associated may have lower overall affinity than epitopes associated with other pathological indications. These observations lend credence to the hypothesis that T1D-associated epitopes may be products of posttranslational modification, and that these modified epitopes are associated with increased HLA binding in peripheral tissue. Indeed, assessment of the binding capacity of PTM versions of known HLA A*02:01 restricted T1D-associated epitopes found increased binding capacity for 44% of the known T-cell epitopes, but only 15% of control peptides. Overall, these findings provide further evidence that human T1D is not solely the result of autoimmunity and may be driven by immune responses to neoantigens, generated from protein processing defects and metabolic dysregulation leading to cell stress.

## Methods

### HLA-peptide binding assays

Peptide-MHC affinities were measured using classical competition assays based on the inhibition of binding of a high affinity radiolabeled ligand to purified HLA class I molecules, as previously described [18]. Briefly, 0.1–1 nM of radiolabeled peptide is co-incubated at room temperature with purified MHC in the presence of a cocktail of protease inhibitors. Following a two-day incubation, MHC bound radioactivity is determined by capturing MHC/peptide complexes on W6/32 (anti-HLA class I) mAb coated Lumitrac 600 plates (Greiner Bio-one, Frickenhausen, Germany), and bound cpm measured using the TopCount (Packard Instrument Co., Meriden, CT) microscintillation counter. The concentration of peptide yielding 50% inhibition of binding of the radiolabeled peptide is calculated. Under the conditions utilized, where [label] < [MHC] and IC50 ≥ [MHC], measured IC50 values are reasonable approximations of true K_d_. Each competitor peptide is tested at six different concentrations covering a 100,000-fold range, and in three or more independent experiments. As a positive control, the unlabeled version of the radiolabeled probe is also tested in each experiment. Utilizing a previously defined threshold [11, 12], peptides with an affinity of 500 nM or better for their restricting allele were defined as high affinity binders; peptides with affinities in the 500–5000 nM range were defined as intermediate binders.

### HLA-peptide binding predictions

Binding predictions were performed using the command-line version of the SMM prediction tool available on the Immune Epitope Database website (http://www.iedb.org) [19, 20]. Besides strong performance in predicting A*02:01 binding capacity, SMM also consistently performs as one of the best prediction tools across a wide array of alleles, and also provides predicted IC50 nM values for all of the alleles considered here. In addition to predicted affinity (IC_50_), the SMM algorithm provides a percentile score expressing the relative capacity of each peptide to bind each specific allele, compared to a universe of potential sequences of the same size.

### Peptide synthesis

Peptides were synthesized by A and A (San Diego) as crude material on a 1 mg scale, or purified (> 95%) by reverse phase HPLC. PTM of individual residues, including deamidation, citrullination, chlorination and oxidation of cysteine was performed as part of the standard Fmoc synthesis. Because of the unavailability of Fmoc-Oxo-His, oxidation of histidine was performed post-synthesis. As a result, histidine cannot be individually oxidized, and in all corresponding peptides all other oxidizable residues (e.g., Cys and Met) are similarly oxidized. Throughout, in the corresponding peptide sequences, the respective PTMs are identified as follows. B: Cysteic acid; J: chloryl tyrosine; O: iso-aspartic acid; Z: iso-glutamic acid; U: citrulline. The human insulin sequence utilized was UniProt accession number P01308.

## Additional files

Additional file 1: Diabetes-associated proteins. Table (Word; .docx) listing antigens associated with T1D, pre-diabetes, and diabetes mellitus studies. (DOCX 56 kb)

Additional file 2: CTL epitopes associated with previously defined diabetes-associated proteins. Table (Word; .docx) listing identified following IEDB query structured as described in the text. (DOCX 131 kb)

Additional file 3: Measured binding affinities. Table (Word; .docx) listing the modified and native peptides studied and their measured HLA A*02:01 binding capacity. (DOCX 135 kb)

Additional file 4: Location of modified residues in full length insulin sequence. Figure (.pdf) highlighting specific insulin residues targeted for modification, as described in the text. (DOCX 103 kb)

## Abbreviations

CTLs: Cytolytic CD8 T-cells
ELISPOT: Enzyme-linked immunospot assay
ER: Endoplasmic reticulum
HLA: Human leukocyte antigen
IC50: Half maximal inhibitory concentration
ICS: Intracellular cytokine staining
IEDB: Immune Epitope Database
MHC: Major histocompatibility
MS: Mass spectrometry
PTM: Post-translational modification
SDS-PAGE: Sodium dodecyl sulfate polyacrylamide gel electrophoresis
T1D: Type 1 diabetes
tTG: Tissue transglutimase
WT: Wild type; amino acids have been abbreviated using standard 3-letter codes.
