# Supplementary material for: Low HLA binding of diabetes-associated CD8+ T-cell epitopes is increased by post translational modifications
Source: BMC Immunol. 2018 Mar 21;19:12. doi: 10.1186/s12865-018-0250-3 (PMC5863483; doi:10.1186/s12865-018-0250-3)
Supplement: Supplementary file 1 — Diabetes-associated proteins. Table (Word; .docx) listing antigens associated with T1D, pre-diabetes, and diabetes mellitus studies. (DOCX 56 kb) [file 12865_2018_250_MOESM1_ESM.docx]

Additional File 1. Diabetes-associated proteins

| **Source Protein** |
| --- |
| AN1-type zinc finger protein 5 |
| Bruton agammaglobulinemia tyrosine kinase |
| Fms-related tyrosine kinase 3 |
| Fms-related tyrosine kinase 3 ligand |
| Glial fibrillary acidic protein isoform 2 |
| Glutamate decarboxylase 2 |
| Heat shock 70 kda protein 1 |
| Heat shock 70 kda protein 6 |
| Heat shock 70 kda protein 6 variant |
| Heat shock 70kda protein 1A variant |
| Heat shock protein HSP 90-beta |
| Insulin |
| Islet amyloid polypeptide precursor |
| Islet-specific glucose-6-phosphatase |
| Islet-specific glucose-6-phosphatase isoform 1 |
| Protein tyrosine phosphatase |
| Tyrosine-protein kinase BTK |
| Tyrosine-protein kinase Lyn isoform B |
| Tyrosine-protein phosphatase non-receptor type 11 |
| Zinc finger protein 36, C3H1 type-like 2 |
| Zinc transporter 8 isoform a |
