# Supplementary material for: Low HLA binding of diabetes-associated CD8+ T-cell epitopes is increased by post translational modifications
Source: BMC Immunol. 2018 Mar 21;19:12. doi: 10.1186/s12865-018-0250-3 (PMC5863483; doi:10.1186/s12865-018-0250-3)
Supplement: Supplementary file 2 — CTL epitopes associated with previously defined diabetes-associated proteins. Table (Word; .docx) listing identified following IEDB query structured as described in the text. (DOCX 131 kb) [file 12865_2018_250_MOESM2_ESM.docx]

**Additional File 2. CTL epitopes associated with previously defined diabetes-associated proteins**

| **Epitope sequence** | **Source Protein** | **Effector Origin^1^** | **Assay Type** | **MHC Restriction** |
| --- | --- | --- | --- | --- |
| SASVQRADTSL | AN1-type zinc finger protein 5 | Direct Ex Vivo | ELISPOT | B7 |
| CLCLLNPQGT | Bruton agammaglobulinemia tyrosine kinase | Direct Ex Vivo | multimer/tetramer | A2 |
| HLASEKVYAI | Bruton agammaglobulinemia tyrosine kinase | Direct Ex Vivo | multimer/tetramer | A2 |
| KLANIQCLCL | Bruton agammaglobulinemia tyrosine kinase | Direct Ex Vivo | multimer/tetramer | A2 |
| KLANIQCPCL | Bruton agammaglobulinemia tyrosine kinase | Direct Ex Vivo | multimer/tetramer | A2 |
| LASEKVYAI | Bruton agammaglobulinemia tyrosine kinase | Direct Ex Vivo | multimer/tetramer | A2 |
| SLTAISTTL | Bruton agammaglobulinemia tyrosine kinase | Direct Ex Vivo | multimer/tetramer | A2 |
| SLTTISTTL | Bruton agammaglobulinemia tyrosine kinase | Direct Ex Vivo | multimer/tetramer | A2 |
| YIPSCTVVGM | Bruton agammaglobulinemia tyrosine kinase | Direct Ex Vivo | multimer/tetramer | A2 |
| KVLHELFGMDI | Fms-related tyrosine kinase 3 | Direct Ex Vivo | multimer/tetramer | A2 |
| VLHELFGMDI | Fms-related tyrosine kinase 3 | Direct Ex Vivo | multimer/tetramer | A2 |
| ALARGAGTVPL | Fms-related tyrosine kinase 3 ligand | Direct Ex Vivo | multimer/tetramer | A2 |
| SMPQGTFPV | Fms-related tyrosine kinase 3 ligand | Direct Ex Vivo | multimer/tetramer | A2 |
| NLAQDLATV | Glial fibrillary acidic protein isoform 2 | Direct Ex Vivo | ELISPOT | A*02:01 |
| QLARQQVHV | Glial fibrillary acidic protein isoform 2 | Direct Ex Vivo | ELISPOT | A*02:01 |
| SLEEEIRFL | Glial fibrillary acidic protein isoform 2 | Direct Ex Vivo | ELISPOT | A*02:01 |
| RFKMFPEVK | Glutamate decarboxylase 2 | In vitro | 51 chromium | A11 |
| MFPEVKEKG | Glutamate decarboxylase 2 | In vitro | 51 chromium | A11 |
| SPGSGFWSF | Glutamate decarboxylase 2 | Direct Ex Vivo | ELISPOT | B*07:02 |
| TSEHSHFSL | Glutamate decarboxylase 2 | In vitro | 51 chromium | B35 |
| ELAEYLYNI | Glutamate decarboxylase 2 | Direct Ex Vivo | ELISPOT | A*02:01 |
| FLQDVMNIL | Glutamate decarboxylase 2 | Direct Ex Vivo | ELISPOT | A*02:01 |
| ILMHCQTTL | Glutamate decarboxylase 2 | Direct Ex Vivo | ELISPOT | A*02:01 |
| LLQEYNWEL | Glutamate decarboxylase 2 | Direct Ex Vivo | ELISPOT | A*02:01 |
| RMMEYGTTMV | Glutamate decarboxylase 2 | Direct Ex Vivo | multimer/tetramer | A*02:01 |
| VMNILLQYV | Glutamate decarboxylase 2 | Direct Ex Vivo | multimer/tetramer | A*02:01 |
| VMNILLQYVV | Glutamate decarboxylase 2 | Direct Ex Vivo | multimer/tetramer | A*02:01 |
| ACDGERPTL | Glutamate decarboxylase 2 | Direct Ex Vivo | ELISPOT | B*07:02 |
| AHVDKCLEL | Glutamate decarboxylase 2 | Direct Ex Vivo | ELISPOT | B*07:02 |
| APVIKARMM | Glutamate decarboxylase 2 | Direct Ex Vivo | ELISPOT | B*07:02 |
| HPRYFNQLST | Glutamate decarboxylase 2 | Direct Ex Vivo | ELISPOT | B*07:02 |
| IPSDLERRIL | Glutamate decarboxylase 2 | Direct Ex Vivo | ELISPOT | B*07:02 |
| LLDVAPLSL | Heat shock 70 kDa protein 1 | Direct Ex Vivo | multimer/tetramer | A*02:01 |
| LLLLDVAPL | Heat shock 70 kDa protein 1 | Direct Ex Vivo | multimer/tetramer | A*02:01 |
| LMGDKSENV | Heat shock 70 kDa protein 1 | ST restimulated | ELISPOT | A*02:01 |
| FIQVYEVERA | Heat shock 70 kDa protein 6 | Direct Ex Vivo | multimer/tetramer | A2 |
| FMTSSWWGA | Heat shock 70 kDa protein 6 | Direct Ex Vivo | multimer/tetramer | A2 |
| FMTSSWWRA | Heat shock 70 kDa protein 6 | Direct Ex Vivo | multimer/tetramer | A2 |
| FMTSSWWRAPL | Heat shock 70 kDa protein 6 | Direct Ex Vivo | multimer/tetramer | A2 |
| GIPPAPHGV | Heat shock 70 kDa protein 6 | Direct Ex Vivo | multimer/tetramer | A2 |
| GLLQVHHSCPL | Heat shock 70 kDa protein 6 | Direct Ex Vivo | multimer/tetramer | A2 |
| GVFIQVYEV | Heat shock 70 kDa protein 6 | Direct Ex Vivo | multimer/tetramer | A2 |
| KCQEVLAWL | Heat shock 70 kDa protein 6 | Direct Ex Vivo | multimer/tetramer | A2 |
| LLGRFELIGI | Heat shock 70 kDa protein 6 | Direct Ex Vivo | multimer/tetramer | A2 |
| LLHVHHSCPL | Heat shock 70 kDa protein 6 | Direct Ex Vivo | multimer/tetramer | A2 |
| LLQVHHSCPL | Heat shock 70 kDa protein 6 | Direct Ex Vivo | multimer/tetramer | A2 |
| NLLGRFELI | Heat shock 70 kDa protein 6 | Direct Ex Vivo | multimer/tetramer | A2 |
| NLLGRFELIGI | Heat shock 70 kDa protein 6 | Direct Ex Vivo | multimer/tetramer | A2 |
| SLASLLPHV | Heat shock 70 kDa protein 6 | Direct Ex Vivo | multimer/tetramer | A2 |
| SMCRFSPLTL | Heat shock 70 kDa protein 6 | Direct Ex Vivo | multimer/tetramer | A2 |
| SVASLLPHV | Heat shock 70 kDa protein 6 | Direct Ex Vivo | multimer/tetramer | A2 |
| VLNSLASLL | Heat shock 70 kDa protein 6 | Direct Ex Vivo | multimer/tetramer | A2 |
| VLNSVASLL | Heat shock 70 kDa protein 6 | Direct Ex Vivo | multimer/tetramer | A2 |
| VLVEGSTRI | Heat shock 70 kDa protein 6 | Direct Ex Vivo | multimer/tetramer | A2 |
| SLFEGVDFYT | Heat shock 70 kDa protein 6 variant | Direct Ex Vivo | multimer/tetramer | A2 |
| GIPPAPRGV | heat shock 70kDa protein 1A variant | Direct Ex Vivo | multimer/tetramer | A2 |
| LIFDLGGGT | heat shock 70kDa protein 1A variant | Direct Ex Vivo | multimer/tetramer | A2 |
| ILDKKVEKV | Heat shock protein HSP 90-beta | Direct Ex Vivo | multimer/tetramer | A*02:01 |
| WGPDPAAA | Insulin | Direct Ex Vivo | ELISPOT | A*02:01 |
| GIVEQCCTSI | Insulin | Direct Ex Vivo | multimer/tetramer | A*02:01 |
| LCGSHLVEAL | Insulin | Direct Ex Vivo | multimer/tetramer | A*02:01 |
| SHLVEALYLV | Insulin | Direct Ex Vivo | multimer/tetramer | A*02:01 |
| ALWGPDPAAA | Insulin | Direct Ex Vivo | multimer/tetramer | A*02:01 |
| HLVEALYLV | Insulin | Direct Ex Vivo | multimer/tetramer | A*02:01 |
| SLYQLENYC | Insulin | Direct Ex Vivo | multimer/tetramer | A*02:01 |
| RLLPLLALL | Insulin | Direct Ex Vivo | multimer/tetramer | A*02:01, A24 |
| VCGERGFFYT | Insulin | Direct Ex Vivo | multimer/tetramer | A*02:01, B8,A1, B18 |
| ALWMRLLPLL | Insulin | Direct Ex Vivo | multimer/tetramer | A*02:01, B8, A24 |
| ALWMRLLPL | Insulin | Direct Ex Vivo | multimer/tetramer | A*02:01, B8 |
| GSHLVEALY | Insulin | Direct Ex Vivo | multimer/tetramer | A1 |
| LVCGERGFFY | Insulin | Direct Ex Vivo | ELISPOT | A1, A3, A11 |
| GERGFFYT | Insulin | Direct Ex Vivo | ELISPOT | A1, B8 |
| LALWGPDPAA | Insulin | Direct Ex Vivo | ELISPOT | A2 |
| RLLPLLALLAL | Insulin | Direct Ex Vivo | ELISPOT | A2 |
| HLCGSHLVEA | Insulin | Direct Ex Vivo | multimer/tetramer | A2 |
| SLQKRGIVEQ | Insulin | Direct Ex Vivo | multimer/tetramer | A2 |
| LYLVCGERGF | Insulin | Direct Ex Vivo | ELISPOT | A24 |
| LWMRLLPLL | Insulin | Direct Ex Vivo | multimer/tetramer | A*24:02 |
| ALWGPDPAAAF | Insulin | Direct Ex Vivo | ELISPOT | A24, A1 |
| ERGFFYTPK | Insulin | Direct Ex Vivo | ELISPOT | A3 |
| PLALEGSLQK | Insulin | Direct Ex Vivo | multimer/tetramer | A3 |
| PLLALLALWG | Insulin | Direct Ex Vivo | multimer/tetramer | A3 |
| ALYLVCGER | Insulin | Direct Ex Vivo | multimer/tetramer | A3, A11 |
| SLQPLALEG | Insulin | Direct Ex Vivo | multimer/tetramer | A3, A2 |
| LPLLALLAL | Insulin | Direct Ex Vivo | multimer/tetramer | B35, B51, B*07:02 |
| WMRLLPLLAL | Insulin | Direct Ex Vivo | multimer/tetramer | B7 |
| FYTPKTRRE | Insulin | Direct Ex Vivo | ELISPOT | B8 |
| FLIVLSVAL | Islet amyloid polypeptide precursor | Direct Ex Vivo | ELISPOT | A*02:01 |
| KLQVFLIVL | Islet amyloid polypeptide precursor | Direct Ex Vivo | multimer/tetramer | A*02:01 |
| FLWSVFMLI | Islet-specific glucose-6-phosphatase | Direct Ex Vivo | ELISPOT | A*02:01 |
| FLWSVFWLI | Islet-specific glucose-6-phosphatase isoform 1 | Direct Ex Vivo | ELISPOT | A*02:01 |
| RLLCALTSL | Islet-specific glucose-6-phosphatase isoform 1 | Direct Ex Vivo | ELISPOT | A*02:01 |
| LNIDLLWSV | Islet-specific glucose-6-phosphatase isoform 1 | Direct Ex Vivo | multimer/tetramer | A*02:01 |
| VLFGLGFAI | Islet-specific glucose-6-phosphatase isoform 1 | Direct Ex Vivo | multimer/tetramer | A*02:01 |
| NLFLFLFAV | Islet-specific glucose-6-phosphatase isoform 1 | Direct Ex Vivo | ELISPOT | A2 |
| YLLLRVLNI | Islet-specific glucose-6-phosphatase isoform 1 | Direct Ex Vivo | ELISPOT | A2 |
| FLFAVGFYL | Islet-specific glucose-6-phosphatase isoform 1 | Direct Ex Vivo | ELISPOT | A*02:01 |
| LLPPLLEHL | Protein tyrosine phosphatase | Direct Ex Vivo | ELISPOT | A*02:01 |
| SLAAGVKLL | Protein tyrosine phosphatase | Direct Ex Vivo | ELISPOT | A*02:01 |
| SLSPLQAEL | Protein tyrosine phosphatase | Direct Ex Vivo | ELISPOT | A*02:01 |
| ALTAVAEEV | Protein tyrosine phosphatase | Direct Ex Vivo | ELISPOT | A*02:01 |
| SLYHVYEVNL | Protein tyrosine phosphatase | Direct Ex Vivo | ELISPOT | A*02:01 |
| TIADFWQMV | Protein tyrosine phosphatase | Direct Ex Vivo | ELISPOT | A*02:01 |
| VIVMLTPLV | Protein tyrosine phosphatase | Direct Ex Vivo | multimer/tetramer | A*02:01 |
| MVWESGCTV | Protein tyrosine phosphatase | Direct Ex Vivo | multimer/tetramer | A2 |
| LASEKVYTI | Tyrosine-protein kinase BTK | Direct Ex Vivo | multimer/tetramer | A2 |
| LMFWSPSHSCA | Tyrosine-protein kinase Lyn isoform B | Direct Ex Vivo | multimer/tetramer | A2 |
| RLQREWHTL | Tyrosine-protein kinase Lyn isoform B | Direct Ex Vivo | multimer/tetramer | A2 |
| RLGPVARTRV | Tyrosine-protein phosphatase non-receptor type 11 | Direct Ex Vivo | multimer/tetramer | A2 |
| STVASRLGPV | Tyrosine-protein phosphatase non-receptor type 11 | Direct Ex Vivo | multimer/tetramer | A2 |
| STVASWLGPV | Tyrosine-protein phosphatase non-receptor type 11 | Direct Ex Vivo | multimer/tetramer | A2 |
| TLSSRVCCRT | Tyrosine-protein phosphatase non-receptor type 11 | Direct Ex Vivo | multimer/tetramer | A2 |
| TVASRLGPV | Tyrosine-protein phosphatase non-receptor type 11 | Direct Ex Vivo | multimer/tetramer | A2 |
| GLPAGAAAQA | Zinc finger protein 36, C3H1 type-like 2 | Direct Ex Vivo | multimer/tetramer | A2 |
| HLSYHRLLPL | Zinc finger protein 36, C3H1 type-like 2 | Direct Ex Vivo | multimer/tetramer | A2 |
| HLSYHWLLPL | Zinc finger protein 36, C3H1 type-like 2 | Direct Ex Vivo | multimer/tetramer | A2 |
| RLLPLWAAL | Zinc finger protein 36, C3H1 type-like 2 | Direct Ex Vivo | multimer/tetramer | A2 |
| RLLPLWAALPL | Zinc finger protein 36, C3H1 type-like 2 | Direct Ex Vivo | multimer/tetramer | A2 |
| RLRPLCCTA | Zinc finger protein 36, C3H1 type-like 2 | Direct Ex Vivo | multimer/tetramer | A2 |
| WLLPLWAAL | Zinc finger protein 36, C3H1 type-like 2 | Direct Ex Vivo | multimer/tetramer | A2 |
| WLLPLWAALPL | Zinc finger protein 36, C3H1 type-like 2 | Direct Ex Vivo | multimer/tetramer | A2 |
| ALGDLFQSI | Zinc transporter 8 isoform a | Direct Ex Vivo | ELISPOT | A*02:01 |
| AVAANIVLTV | Zinc transporter 8 isoform a | Direct Ex Vivo | ELISPOT | A*02:01 |
| FLLSLFSLWL | Zinc transporter 8 isoform a | Direct Ex Vivo | ELISPOT | A*02:01 |
| HIAGSLAVV | Zinc transporter 8 isoform a | Direct Ex Vivo | ELISPOT | A*02:01 |
| ILAVDGVLSV | Zinc transporter 8 isoform a | Direct Ex Vivo | ELISPOT | A*02:01 |
| ILKDFSILL | Zinc transporter 8 isoform a | Direct Ex Vivo | ELISPOT | A*02:01 |
| ILVLASTITI | Zinc transporter 8 isoform a | Direct Ex Vivo | ELISPOT | A*02:01 |
| IQATVMIIV | Zinc transporter 8 isoform a | Direct Ex Vivo | ELISPOT | A*02:01 |
| KMYAFTLES | Zinc transporter 8 isoform a | Direct Ex Vivo | ELISPOT | A*02:01 |
| RLLYPDYQI | Zinc transporter 8 isoform a | Direct Ex Vivo | ELISPOT | A*02:01 |
| SISVLISAL | Zinc transporter 8 isoform a | Direct Ex Vivo | ELISPOT | A*02:01 |
| TMHSLTIQM | Zinc transporter 8 isoform a | Direct Ex Vivo | ELISPOT | A*02:01 |
| VAANIVLTV | Zinc transporter 8 isoform a | Direct Ex Vivo | ELISPOT | A*02:01 |
| VVTGVLVYL | Zinc transporter 8 isoform a | Direct Ex Vivo | ELISPOT | A*02:01 |
| LLIDLTSFL | Zinc transporter 8 isoform a | Cell Line / Clone | ELISPOT | A*02:01 |
| LLSILCIWV | Zinc transporter 8 isoform a | Cell Line / Clone | ELISPOT | A*02:01 |
| LLSLFSLWL | Zinc transporter 8 isoform a | Cell Line / Clone | ELISPOT | A*02:01 |

1. “Effector origin” and “Assay type” are representative of the experimental data available for each specific epitope, and reflects the most stringent experimental conditions reported, where “direct ex vivo” > “short-term restimulated” > “in vitro” > “cell line/clone”, and “multimer/tetramer” > “ELISPOT” > “ICS” > “51 chromium”. See [[7](#_ENREF_7)]
