# Supplementary material for: Low HLA binding of diabetes-associated CD8+ T-cell epitopes is increased by post translational modifications
Source: BMC Immunol. 2018 Mar 21;19:12. doi: 10.1186/s12865-018-0250-3 (PMC5863483; doi:10.1186/s12865-018-0250-3)
Supplement: Supplementary file 3 — Measured binding affinities. Table (Word; .docx) listing the modified and native peptides studied and their measured HLA A*02:01 binding capacity. (DOCX 135 kb) [file 12865_2018_250_MOESM3_ESM.docx]

Additional File 3. Measured binding affinities

| **Insulin** |  | **Unmodified** |  | **Reported** | **Modified** |  | **A*02:01 binding (IC50 nM)** | |
| --- | --- | --- | --- | --- | --- | --- | --- | --- |
| **start** | **Segment** | **sequence** | **Len** | **A*02 epitope** | **sequence*** | **Modification(s)** | **Modified** | **WT** |
| 1 | Leader | MALWMRLLP | 9 |  | MALWMULLP | Citrullination | 2117 | 4539 |
| 1 | Leader | MALWMRLLPL | 10 |  | MALWMULLPL | Citrullination | 262 | 1255 |
| 2 | Leader | ALWMRLLPL | 9 | Yes | ALWMULLPL | Citrullination | 28 | 218 |
| 2 | Leader | ALWMRLLPLL | 10 | Yes | ALWMULLPLL | Citrullination | 69 | 190 |
| 3 | Leader | LWMRLLPLL | 9 |  | LWMULLPLL | Citrullination | 148 | 266 |
| 3 | Leader | LWMRLLPLLA | 10 |  | LWMULLPLLA | Citrullination | 61 | 406 |
| 4 | Leader | WMRLLPLLA | 9 |  | WMULLPLLA | Citrullination | 1007 | 1530 |
| 4 | Leader | WMRLLPLLAL | 10 |  | WMULLPLLAL | Citrullination | 310 | 654 |
| 5 | Leader | MRLLPLLAL | 9 |  | MULLPLLAL | Citrullination | 1863 | 228 |
| 5 | Leader | MRLLPLLALL | 10 |  | MULLPLLALL | Citrullination | 98 | 10 |
| 6 | Leader | RLLPLLALL | 9 | Yes | ULLPLLALL | Citrullination | 83 | 145 |
| 6 | Leader | RLLPLLALLA | 10 |  | ULLPLLALLA | Citrullination | 70 | 11 |
| 25 | B chain | FVNQHLCGS | 9 |  | FVNQHLBGS | Oxidation of C | - | - |
| 25 | B chain | FVNQHLCGS | 9 |  | FVDEHLCGS | Deamidation | - | - |
| 25 | B chain | FVNQHLCGS | 9 |  | FVOZHLCGS | Deamidation | - | - |
| 25 | B chain | FVNQHLCGS | 9 |  | YVNQXLBGS | Pan oxidation | - | - |
| 25 | B chain | FVNQHLCGS | 9 |  | FVNQXLCGS | Pan oxidation | - | - |
| 25 | B chain | FVNQHLCGSH | 10 |  | FVNQHLBGSH | Oxidation of C | - | 13213 |
| 25 | B chain | FVNQHLCGSH | 10 |  | FVDEHLCGSH | Deamidation | - | 13213 |
| 25 | B chain | FVNQHLCGSH | 10 |  | FVOZHLCGSH | Deamidation | - | 13213 |
| 25 | B chain | FVNQHLCGSH | 10 |  | YVNQXLBGSX | Pan oxidation | - | 13213 |
| 25 | B chain | FVNQHLCGSH | 10 |  | FVNQXLCGSH | Pan oxidation | - | 13213 |
| 26 | B chain | VNQHLCGSH | 9 |  | VNQHLBGSH | Oxidation of C | - | - |
| 26 | B chain | VNQHLCGSH | 9 |  | VDEHLCGSH | Deamidation | - | - |
| 26 | B chain | VNQHLCGSH | 9 |  | VOZHLCGSH | Deamidation | - | - |
| 26 | B chain | VNQHLCGSH | 9 |  | VNQXLBGSX | Pan oxidation | - | - |
| 26 | B chain | VNQHLCGSH | 9 |  | VNQXLCGSH | Pan oxidation | - | - |
| 26 | B chain | VNQHLCGSHL | 10 |  | VNQHLBGSHL | Oxidation of C | - | 6643 |
| 26 | B chain | VNQHLCGSHL | 10 |  | VDEHLCGSHL | Deamidation | - | 6643 |
| 26 | B chain | VNQHLCGSHL | 10 |  | VOZHLCGSHL | Deamidation | - | 6643 |
| 26 | B chain | VNQHLCGSHL | 10 |  | VNQXLBGSXL | Pan oxidation | - | 6643 |
| 26 | B chain | VNQHLCGSHL | 10 |  | VNQXLCGSHL | Pan oxidation | - | 6643 |
| 27 | B chain | NQHLCGSHL | 9 |  | NQHLBGSHL | Oxidation of C | - | - |
| 27 | B chain | NQHLCGSHL | 9 |  | DEHLCGSHL | Deamidation | - | - |
| 27 | B chain | NQHLCGSHL | 9 |  | OZHLCGSHL | Deamidation | - | - |
| 27 | B chain | NQHLCGSHL | 9 |  | NQXLBGSXL | Pan oxidation | - | - |
| 27 | B chain | NQHLCGSHL | 9 |  | NQXLCGSHL | Pan oxidation | - | - |
| 27 | B chain | NQHLCGSHLV | 10 |  | NQHLBGSHLV | Oxidation of C | - | 7994 |
| 27 | B chain | NQHLCGSHLV | 10 |  | DEHLCGSHLV | Deamidation | - | 7994 |
| 27 | B chain | NQHLCGSHLV | 10 |  | OZHLCGSHLV | Deamidation | - | 7994 |
| 27 | B chain | NQHLCGSHLV | 10 |  | NQXLBGSXLV | Pan oxidation | - | 7994 |
| 27 | B chain | NQHLCGSHLV | 10 |  | NQXLCGSHLV | Pan oxidation | - | 7994 |
| 28 | B chain | QHLCGSHLV | 9 |  | EHLCGSHLV | Deamidation | 7237 | - |
| 28 | B chain | QHLCGSHLV | 9 |  | QHLBGSHLV | Oxidation of C | 37883 | - |
| 28 | B chain | QHLCGSHLV | 9 |  | QXLBGSXLV | Pan oxidation | - | - |
| 28 | B chain | QHLCGSHLV | 9 |  | QXLCGSHLV | Pan oxidation | - | - |
| 28 | B chain | QHLCGSHLV | 9 |  | ZHLCGSHLV | Deamidation | - | - |
| 28 | B chain | QHLCGSHLVE | 10 |  | QHLBGSHLVE | Oxidation of C | - | - |
| 28 | B chain | QHLCGSHLVE | 10 |  | EHLCGSHLVE | Deamidation | - | - |
| 28 | B chain | QHLCGSHLVE | 10 |  | QXLBGSXLVE | Pan oxidation | - | - |
| 28 | B chain | QHLCGSHLVE | 10 |  | QXLCGSHLVE | Pan oxidation | - | - |
| 28 | B chain | QHLCGSHLVE | 10 |  | ZHLCGSHLVE | Deamidation | - | - |
| 29 | B chain | HLCGSHLVE | 9 |  | HLBGSHLVE | Oxidation of C | - | - |
| 29 | B chain | HLCGSHLVE | 9 |  | XLBGSXLVE | Pan oxidation | - | - |
| 29 | B chain | HLCGSHLVE | 9 |  | XLCGSHLVE | Pan oxidation | - | - |
| 29 | B chain | HLCGSHLVEA | 10 | Yes | HLBGSHLVEA | Oxidation of C | 6210 | 7536 |
| 29 | B chain | HLCGSHLVEA | 10 | Yes | XLBGSXLVEA | Pan oxidation | 10687 | 7536 |
| 29 | B chain | HLCGSHLVEA | 10 | Yes | XLCGSHLVEA | Pan oxidation | 11800 | 7536 |
| 30 | B chain | LCGSHLVEA | 9 |  | LBGSHLVEA | Oxidation of C | 14796 | 1353 |
| 30 | B chain | LCGSHLVEA | 9 |  | LBGSXLVEA | Pan oxidation | 13270 | 1353 |
| 30 | B chain | LCGSHLVEAL | 10 | Yes | LBGSHLVEAL | Oxidation of C | - | - |
| 30 | B chain | LCGSHLVEAL | 10 | Yes | LBGSXLVEAL | Pan oxidation | - | - |
| 31 | B chain | CGSHLVEAL | 9 |  | BGSHLVEAL | Oxidation of C | - | - |
| 31 | B chain | CGSHLVEAL | 9 |  | BGSXLVEAL | Pan oxidation | - | - |
| 31 | B chain | CGSHLVEALY | 10 |  | BGSHLVEALY | Oxidation of C | 8043 | 33691 |
| 31 | B chain | CGSHLVEALY | 10 |  | CGSHLVEALJ | Chlorination | 38276 | 33691 |
| 31 | B chain | CGSHLVEALY | 10 |  | BGSXLVEALY | Pan oxidation | - | 33691 |
| 32 | B chain | GSHLVEALY | 9 |  | GSHLVEALJ | Chlorination | 30634 | 3962 |
| 32 | B chain | GSHLVEALYL | 10 |  | GSHLVEALJL | Chlorination | 6837 | 1770 |
| 33 | B chain | SHLVEALYL | 9 |  | SHLVEALJL | Chlorination | 445 | 257 |
| 33 | B chain | SHLVEALYLV | 10 | Yes | SHLVEALJLV | Chlorination | 1066 | 2153 |
| 34 | B chain | HLVEALYLV | 9 | Yes | HLVEALJLV | Chlorination | 55 | 134 |
| 34 | B chain | HLVEALYLVC | 10 |  | HLVEALJLVC | Chlorination | 2132 | 692 |
| 35 | B chain | LVEALYLVC | 9 |  | LVEALJLVC | Chlorination | 11768 | 11770 |
| 35 | B chain | LVEALYLVCG | 10 |  | LVEALJLVCG | Chlorination | 15300 | 23250 |
| 36 | B chain | VEALYLVCG | 9 |  | VEALJLVCG | Chlorination | 18759 | 14340 |
| 36 | B chain | VEALYLVCGE | 10 |  | VEALJLVCGE | Chlorination | - | 8867 |
| 37 | B chain | EALYLVCGE | 9 |  | EALJLVCGE | Chlorination | 13342 | 12021 |
| 37 | B chain | EALYLVCGER | 10 |  | EALYLVCGEU | Citrullination | 3100 | 10069 |
| 37 | B chain | EALYLVCGER | 10 |  | EALJLVCGER | Chlorination | 8562 | 10069 |
| 38 | B chain | ALYLVCGER | 9 |  | ALJLVCGER | Chlorination | 2988 | 1792 |
| 38 | B chain | ALYLVCGER | 9 |  | ALYLVCGEU | Citrullination | 1241 | 1792 |
| 38 | B chain | ALYLVCGERG | 10 |  | ALJLVCGERG | Chlorination | 2734 | 8784 |
| 38 | B chain | ALYLVCGERG | 10 |  | ALYLVCGEUG | Citrullination | 9076 | 8784 |
| 39 | B chain | LYLVCGERG | 9 |  | LYLVCGEUG | Citrullination | 12493 | 28006 |
| 39 | B chain | LYLVCGERG | 9 |  | LJLVCGERG | Chlorination | 19307 | 28006 |
| 39 | B chain | LYLVCGERGF | 10 |  | LJLVCGERGF | Chlorination | 14255 | 13418 |
| 39 | B chain | LYLVCGERGF | 10 |  | LYLVCGEUGF | Citrullination | 18784 | 13418 |
| 39 | B chain | LYLVCGERGF | 10 |  | LYLVCGERGY | Oxidation of F | - | 13418 |
| 39 | B chain | LYLVCGERGF | 10 |  | LYLVBGERGY | Pan oxidation | 35512 | 13418 |
| 40 | B chain | YLVCGERGF | 9 |  | JLVCGERGF | Chlorination | 3163 | 6742 |
| 40 | B chain | YLVCGERGF | 9 |  | YLVBGERGY | Pan oxidation | 3244 | 6742 |
| 40 | B chain | YLVCGERGF | 9 |  | YLVCGEUGF | Citrullination | 11365 | 6742 |
| 40 | B chain | YLVCGERGF | 9 |  | YLVCGERGY | Oxidation of F | 10814 | 6742 |
| 40 | B chain | YLVCGERGFF | 10 |  | JLVCGERGFF | Chlorination | 1690 | 1399 |
| 40 | B chain | YLVCGERGFF | 10 |  | YLVCGEUGFF | Citrullination | 2617 | 1399 |
| 40 | B chain | YLVCGERGFF | 10 |  | YLVCGERGYF | Oxidation of F | 2009 | 1399 |
| 40 | B chain | YLVCGERGFF | 10 |  | YLVBGERGYY | Pan oxidation | 2421 | 1399 |
| 41 | B chain | LVCGERGFF | 9 |  | LVCGEUGFF | Citrullination | - | - |
| 41 | B chain | LVCGERGFF | 9 |  | LVCGERGYF | Oxidation of F | - | - |
| 41 | B chain | LVCGERGFF | 9 |  | LVBGERGYY | Pan oxidation | - | - |
| 41 | B chain | LVCGERGFFY | 10 |  | LVCGERGFFJ | Chlorination | 27675 | 23164 |
| 41 | B chain | LVCGERGFFY | 10 |  | LVCGEUGFFY | Citrullination | - | 23164 |
| 41 | B chain | LVCGERGFFY | 10 |  | LVCGERGYFY | Oxidation of F | - | 23164 |
| 41 | B chain | LVCGERGFFY | 10 |  | LVBGERGYYY | Pan oxidation | - | 23164 |
| 42 | B chain | VCGERGFFY | 9 |  | VCGERGFFJ | Chlorination | - | - |
| 42 | B chain | VCGERGFFY | 9 |  | VCGEUGFFY | Citrullination | 33476 | - |
| 42 | B chain | VCGERGFFY | 9 |  | VCGERGYFY | Oxidation of F | - | - |
| 42 | B chain | VCGERGFFY | 9 |  | VBGERGYYY | Pan oxidation | - | - |
| 42 | B chain | VCGERGFFYT | 10 | Yes | VCGERGFFJT | Chlorination | - | - |
| 42 | B chain | VCGERGFFYT | 10 | Yes | VCGEUGFFYT | Citrullination | - | - |
| 42 | B chain | VCGERGFFYT | 10 | Yes | VCGERGYFYT | Oxidation of F | - | - |
| 42 | B chain | VCGERGFFYT | 10 | Yes | VBGERGYYYT | Pan oxidation | - | - |
| 43 | B chain | CGERGFFYT | 9 |  | CGERGFFJT | Chlorination | 38400 | - |
| 43 | B chain | CGERGFFYT | 9 |  | CGEUGFFYT | Citrullination | - | - |
| 43 | B chain | CGERGFFYT | 9 |  | CGERGYFYT | Oxidation of F | 26255 | - |
| 43 | B chain | CGERGFFYT | 9 |  | BGERGYYYT | Pan oxidation | - | - |
| 43 | B chain | CGERGFFYTP | 10 |  | CGERGFFJTP | Chlorination | 27807 | - |
| 43 | B chain | CGERGFFYTP | 10 |  | CGEUGFFYTP | Citrullination | - | - |
| 43 | B chain | CGERGFFYTP | 10 |  | CGERGYFYTP | Oxidation of F | - | - |
| 43 | B chain | CGERGFFYTP | 10 |  | BGERGYYYTP | Pan oxidation | - | - |
| 44 | B chain | GERGFFYTP | 9 |  | GERGFFJTP | Chlorination | - | - |
| 44 | B chain | GERGFFYTP | 9 |  | GEUGFFYTP | Citrullination | - | - |
| 44 | B chain | GERGFFYTP | 9 |  | GERGYFYTP | Oxidation of F | - | - |
| 44 | B chain | GERGFFYTP | 9 |  | GERGYYYTP | Pan oxidation | - | - |
| 44 | B chain | GERGFFYTPK | 10 |  | GERGFFJTPK | Chlorination | - | - |
| 44 | B chain | GERGFFYTPK | 10 |  | GEUGFFYTPK | Citrullination | - | - |
| 44 | B chain | GERGFFYTPK | 10 |  | GERGYFYTPK | Oxidation of F | - | - |
| 44 | B chain | GERGFFYTPK | 10 |  | GERGYYYTPK | Pan oxidation | - | - |
| 45 | B chain | ERGFFYTPK | 9 |  | ERGFFJTPK | Chlorination | - | - |
| 45 | B chain | ERGFFYTPK | 9 |  | EUGFFYTPK | Citrullination | 38093 | - |
| 45 | B chain | ERGFFYTPK | 9 |  | ERGYFYTPK | Oxidation of F | - | - |
| 45 | B chain | ERGFFYTPK | 9 |  | ERGYYYTPK | Pan oxidation | - | - |
| 45 | B chain | ERGFFYTPKT | 10 |  | ERGYYYTPKT | Pan oxidation | 5730 | - |
| 45 | B chain | ERGFFYTPKT | 10 |  | ERGFFJTPKT | Chlorination | - | - |
| 45 | B chain | ERGFFYTPKT | 10 |  | EUGFFYTPKT | Citrullination | 36308 | - |
| 45 | B chain | ERGFFYTPKT | 10 |  | ERGYFYTPKT | Oxidation of F | - | - |
| 46 | B chain | RGFFYTPKT | 9 |  | RGYYYTPKT | Pan oxidation | 8805 | - |
| 46 | B chain | RGFFYTPKT | 9 |  | RGFFJTPKT | Chlorination | - | - |
| 46 | B chain | RGFFYTPKT | 9 |  | UGFFYTPKT | Citrullination | 27300 | - |
| 46 | B chain | RGFFYTPKT | 9 |  | RGYFYTPKT | Oxidation of F | - | - |
| 46 |  | RGFFYTPKTR | 10 |  | UGFFYTPKTU | Citrullination | - | - |
| 47 |  | GFFYTPKTR | 9 |  | GFFYTPKTU | Citrullination | 38420 | 12176 |
| 47 |  | GFFYTPKTRR | 10 |  | GFFYTPKTUU | Citrullination | - | - |
| 48 |  | FFYTPKTRR | 9 |  | FFYTPKTUU | Citrullination | - | - |
| 48 |  | FFYTPKTRRE | 10 |  | FFYTPKTUUE | Citrullination | 28285 | - |
| 49 |  | FYTPKTRRE | 9 |  | FYTPKTUUE | Citrullination | - | - |
| 49 |  | FYTPKTRREA | 10 |  | FYTPKTUUEA | Citrullination | - | - |
| 50 |  | YTPKTRREA | 9 |  | YTPKTUUEA | Citrullination | 34672 | - |
| 50 |  | YTPKTRREAE | 10 |  | YTPKTUUEAE | Citrullination | - | - |
| 51 |  | TPKTRREAE | 9 |  | TPKTUUEAE | Citrullination | - | - |
| 51 |  | TPKTRREAED | 10 |  | TPKTUUEAED | Citrullination | - | - |
| 52 |  | PKTRREAED | 9 |  | PKTUUEAED | Citrullination | - | - |
| 52 |  | PKTRREAEDL | 10 |  | PKTUUEAEDL | Citrullination | - | - |
| 53 |  | KTRREAEDL | 9 |  | KTUUEAEDL | Citrullination | - | - |
| 53 |  | KTRREAEDLQ | 10 |  | KTUUEAEDLQ | Citrullination | 22304 | - |
| 54 |  | TRREAEDLQ | 9 |  | TUUEAEDLQ | Citrullination | - | - |
| 54 |  | TRREAEDLQV | 10 |  | TUUEAEDLQV | Citrullination | 30243 | - |
| 55 |  | RREAEDLQV | 9 |  | UUEAEDLQV | Citrullination | - | - |
| 55 |  | RREAEDLQVG | 10 |  | UUEAEDLQVG | Citrullination | - | - |
| 56 |  | REAEDLQVG | 9 |  | UEAEDLQVG | Citrullination | - | - |
| 56 |  | REAEDLQVGQ | 10 |  | UEAEDLQVGQ | Citrullination | - | 23725 |
| 80 |  | LALEGSLQKR | 10 |  | LALEGSLQKU | Citrullination | 16354 | - |
| 81 |  | ALEGSLQKR | 9 |  | ALEGSLQKU | Citrullination | - | - |
| 81 |  | ALEGSLQKRG | 10 |  | ALEGSLQKUG | Citrullination | - | - |
| 82 |  | LEGSLQKRG | 9 |  | LEGSLQKUG | Citrullination | - | - |
| 82 |  | LEGSLQKRGI | 10 |  | LEGSLQKUGI | Citrullination | - | - |
| 83 |  | EGSLQKRGI | 9 |  | EGSLQKUGI | Citrullination | - | - |
| 83 |  | EGSLQKRGIV | 10 |  | EGSLQKUGIV | Citrullination | - | - |
| 84 |  | GSLQKRGIV | 9 |  | GSLQKUGIV | Citrullination | - | 32748 |
| 84 |  | GSLQKRGIVE | 10 |  | GSLQKUGIVE | Citrullination | 34549 | - |
| 85 |  | SLQKRGIVE | 9 |  | SLQKUGIVE | Citrullination | - | 5952 |
| 85 |  | SLQKRGIVEQ | 10 | Yes | SLQKUGIVEQ | Citrullination | - | - |
| 86 |  | LQKRGIVEQ | 9 |  | LQKUGIVEQ | Citrullination | - | - |
| 86 |  | LQKRGIVEQC | 10 |  | LQKUGIVEQC | Citrullination | - | - |
| 87 |  | QKRGIVEQC | 9 |  | QKUGIVEQC | Citrullination | - | - |
| 87 |  | QKRGIVEQCC | 10 |  | QKUGIVEQCC | Citrullination | - | - |
| 88 |  | KRGIVEQCC | 9 |  | KUGIVEQCC | Citrullination | - | - |
| 88 |  | KRGIVEQCCT | 10 |  | KUGIVEQCCT | Citrullination | - | - |
| 89 |  | RGIVEQCCT | 9 |  | UGIVEQCCT | Citrullination | - | - |
| 89 |  | RGIVEQCCTS | 10 |  | UGIVEQCCTS | Citrullination | - | - |
| Modified residues are identified as follows: B: Cysteic acid; J: chloryl tyrosine; O: iso-aspartic acid; Z: iso-glutamic acid; U: citrulline. | | | | | | | | |
