# Supplementary material for: Low HLA binding of diabetes-associated CD8+ T-cell epitopes is increased by post translational modifications
Source: BMC Immunol. 2018 Mar 21;19:12. doi: 10.1186/s12865-018-0250-3 (PMC5863483; doi:10.1186/s12865-018-0250-3)
Supplement: Supplementary file 4 — Location of modified residues in full length insulin sequence. Figure (.pdf) highlighting specific insulin residues targeted for modification, as described in the text. (DOCX 103 kb) [file 12865_2018_250_MOESM4_ESM.docx]

**Additional File 4. Location of modified residues in full length insulin sequence**
